# Synthesis and Characterization of Polyimide with High Blackness and Low Thermal Expansion by Introducing 3,6-bis(thiophen-2-yl)diketopyrrolopyrrole-Based Chromophores

**DOI:** 10.3390/polym16233365

**Published:** 2024-11-29

**Authors:** Yiwu Liu, Xueyuan Liu, Jinghua Tan, Jie Huang, Jiazhen Yuan, Huipeng Li, Jieping Guo, Penghao Yu, Yue Chen

**Affiliations:** National and Local Joint Engineering Research Center for Advanced Packaging Materials and Technology, Key Laboratory of Advanced Packaging Materials and Technology of Hunan Province, School of Packaging and Materials Engineering, Hunan University of Technology, Zhuzhou 412007, China

**Keywords:** black polyimide, chromophore, 3,6-bis(thiophen-2-yl)diketopyrrolopyrroles, complete absorption of visible light, low coefficient of thermal expansion

## Abstract

The market demand for black polyimide (BPI) has grown hugely in the field of flexible copper-clad laminates (FCCLs) as a replacement for transparent yellow polyimide. The 3,6-bis(thiophen-2-yl)diketopyrrolopyrroles (TDPP) derivative is recognized for its high molar extinction coefficient. In this research, we have synthesized a diamine monomer named 3,6-bis[5-(4-amino-3-fluorophenyl)thiophen-2-yl]-2,5-bis(2-ethylhexyl)pyrrolo[4,3-c]pyrrole-1,4-dione (DPPTENFPDA), featuring a TDPP unit attached by fluorinated benzene rings. The subsequent reaction of DPPTENFPDA with pyromellitic dianhydride (PMDA) yielded an inherent BPI (DPPTENFPPI). By introducing chromophores derived from TDPP, the light absorption spectrum of DPPTENFPPI was broadened and red-shifted, thereby achieving full absorption within the visible spectrum and producing a highly black color that has a cut-off wavelength (*λ_cut_*) of 717 nm and a CIE-Lab coordinate L* of 0.86. Additionally, DPPTENFPPI exhibited a low coefficient of thermal expansion (CTE) and remarkable thermal and electrical performance. Density functional theory calculations were conducted to explore the electronic nature of DPPTENFPPI. The outcomes revealed that the excellent light absorption of DPPTENFPPI predominantly originates from the transition from HOMO to LUMO + 1 within the chromophore moiety. The FCCL made from DPPTENFPPI films has high solder heat resistance and peel strength. This research contributes valuable insights into the structure and design of high-performance intrinsically black PIs for microelectronics applications.

## 1. Introduction

Due to their exceptional mechanical, thermal, radiation resistance, and electrical insulation properties, polyimides (PIs) have found extensive use in optoelectronics, microelectronics, gas separation, aerospace, and other fields [1,2]. In particular, PI is frequently used in FCCLs, which are crucial building blocks for optoelectronic devices [3,4,5]. Owing to its transparent yellow appearance, conventional Kapton film has less ability to conceal the underlying circuit on the copper foil (Figure S1a) [6]. To solve this problem, a unique PI that can absorb visible light, known as black PI (BPI), is used in FCCLs (Figure S1b) [7]. The BPI films in FCCLs not only safeguard the intellectual properties of circuit design but also prevent the copper foil from oxidation under light. Moreover, BPI film also possesses widespread application in heat-resistant labels, coatings for lithium batteries, wireless charging, and audio speaker components [3].

In recent years, adding black filler to a polyimide matrix has become the most popular technique utilized in the production of BPIs. Carbon-based fillers [8,9], metal oxide [10], or alternative organic [11] and inorganic black fillers [12] are the most commonly used fillers. However, due to the agglomeration and inherent conductivity of fillers, PI composites that incorporate inorganic fillers exhibit a reduction in mechanical and electrical performance [7]. Additionally, because of the low thermostability of organic black fillers, the BPI composites that originate from them often show a noticeable reduction in thermal performance [13]. These drawbacks severely restrict the use of BPI composites.

To overcome these problems, researchers have made an effort to prepare intrinsic BPIs that can retain favorable electrical and thermal characteristics. Currently, there are two basic methods for creating intrinsic black PIs. One involves improving the creation of charge transfer complexes (CTCs) in the PI matrix, while the other involves introducing chromophores to the PI matrix [14]. For instance, Liu created BPIs by increasing the formation of CTCs, but the resulting BPIs only showed a *λ_cut_* of 555 nm [15]. For achieving total visible spectrum absorption, the *λ_cut_* needs to surpass 700 nm [7]. Hence, the obtained PI did not achieve total visible spectrum absorption, thus failing to present an ideal black appearance. In our previous research, tetraphenylcyclopentadienone (TPCP) chromophores were incorporated to broaden the visible absorption of PIs [14]. The resulting BPI film achieved a high *λ_cut_* of 684 nm. However, BPI film has a large CTE of 51 ppm/K, which is significantly greater than the CTE of copper foils (18 ppm/K) [3]. Such a disparity in CTE values between the polymeric component and the copper foil layer will cause cracking, warping, and delamination within the final FCCLs. Thus, it is urgently necessary to develop black PIs that are characterized by a low CTE value and total visible spectrum absorption.

**Scheme 1:**
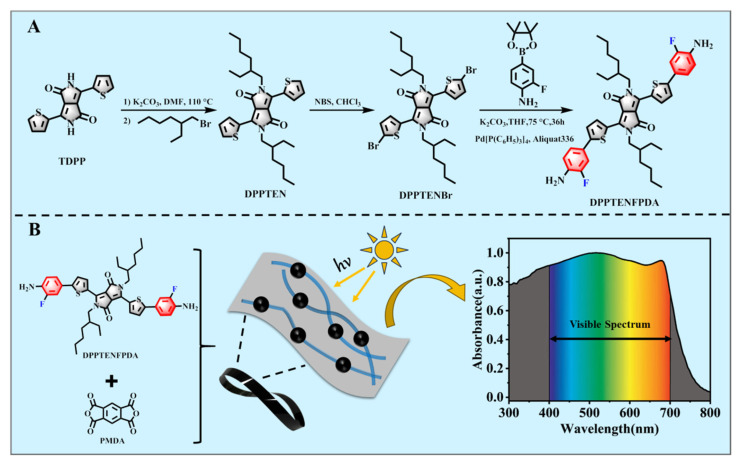
Synthesis route of DPPTENFPDA (**A**) and the coloration mechanism of DPPTENFPPI (**B**).

TDPP is a typical chromophore molecule characterized by a conjugated planar structure [16]. It possesses flexible structure tunability and a high molar extinction coefficient of 3 × 10^4^ M^−1^·cm^−1^ [17]. In this study, a black PI was obtained by introducing a chromophore system based on a TDPP moiety. As illustrated in Scheme 1, a diamine compound (DPPTENFPDA) containing TDPP-derived chromophores was successfully synthesized, which was then polymerized with PMDA to form PI (DPPTENFPPI). In DPPTENFPDA, the TDPP moiety is linked to two fluorinated benzene rings, thus forming a big chromophore. Based on the principles of auxochromes and chromophores, the attachment of the fluorinated benzene ring to the TDPP extends the π-conjugation framework and enhances the motion of electrons along the π-bonds system, which, in turn, reduces the band-gap energy. This molecular design is advantageous for broadening and red-shifting the absorption spectra of PIs, thereby enabling total visible spectrum absorption. In addition, the incorporation of 2-ethylhexyl groups into the TDPP improves the solubility of DPPTENFPDA and guarantees its successful polycondensation with dianhydride PMDA to generate PIs with high molecular weight. Furthermore, the rigidity and planarity of the chromophore system are beneficial for improving the thermal performance and dimensional stability of PI. These positive properties were confirmed through a series of characterization methods. The optical, thermal, and electrical characteristics of DPPTENFPPI were systemically analyzed and compared with conventional Kapton film derived from PMDA and 4,4′-oxydianiline (ODA). In addition, quantum chemical calculations were conducted to elucidate the distinctive black coloration of DPPTENFPPI. This research has implications for the advancement of black PIs with excellent comprehensive properties tailored for microelectronics applications. 

## 2. Experimental Section

### 2.1. Materials

TDPP, potassium carbonate, 2-ethylhexyl bromide, *N*-bromosuccinimide, magnesium sulfate (MgSO_4_), 4-amino-3-fluorophenylboronic acid pinacol ester, Aliquat 336, Pd[P(C_6_H_5_)_3_]_4_, PMDA, and ODA were obtained from Alfa-Aesar. Before usage, the PMDA was vacuum-heated for six hours at 110 °C. Dichloromethane, tetrahydrofuran (THF), n-hexane, 1-methyl-2-pyrrolidinone (NMP), ethyl acetate, chloroform, and N,N-dimethylformamide (DMF) were obtained from the Sinopharm Chemical Reagent Company (Shanghai, China). These reagents were utilized as supplied, with no additional purification.

### 2.2. Instrumentation

The Bruker ARX400 MHz instrument was adopted to test each nuclear magnetic resonance spectrum (NMR). Tetramethylsilane (TMS) was used as an internal reference. The sample solution was made from 5~15 mg of chemical in 0.5 mL of deuterated chloroform (CDCl_3_). The Thermo Scientific ISQ-7000 (Thermo Fisher Scientific, Waltham, MA, USA) and AcquityUPLC/UPC2/Xevo G2-XS QTOFMS instruments (Waters Corporation, Milford, USA) were used to measure the mass spectra (MS) of the intermediates and diamine, respectively. The Vario EL cube element analyzer instrument (Elementar Analysen systeme GmbH, Langenselbold, Germany) was adopted for element analysis. The Nicolet iS10 FTIR instrument (Thermo Fisher Scientific, Waltham, MA, USA) was adopted to obtain the Fourier-transform infrared spectra (FTIR). A multi-angle laser light-scattering system (Wyatt Technology Corporation, Santa Barbara, CA, USA) was utilized to test the molecular weight of polyamic acid (PAA) with the eluent of DMF at 50 °C. A thermal analyzer (DMA 850, TA Instruments, New Castle, DE, USA) was adopted to test the dynamic mechanical spectra (DMA) of the PI films, using a constant frequency of 1 Hz in tensile mode. The thermal expansion coefficient of the PI film was measured using thermal mechanical analysis (TMA Q400, TA Instruments, New Castle, DE, USA). Hioki sheet sample electrodes (SME 8310, Hioki, Ueda, Japan) were used to measure the volume and surface resistivity of PI. A ZXIBV-2/10 breakdown voltage instrument (Guilin Zhangxin Measuring Instrument Corporation, Guilin, China) was adopted for the dielectric strength test. The test voltage and ramp-up rate were 10 kV and 0.5 kV/s, respectively. The test was carried out at 20 °C under a relative humidity of 50%. The UV-vis transmittance and the absorption spectrum were recorded on a PerkinElmer UV-vis instrument (Lambda 950, PerkinElmer, Waltham, MA, USA). A PI film with a thickness of 20 μm was used for the test. A scanning electron microscope (ZE018, SEM, Zeiss, Oberkochen, Germany) was employed to study the surface and section morphology of the PI film.

An X-rite eXact spectrophotometer (X-Rite Corporation, Grand Rapids, MI, USA) was used to measure the Commission International Eclairage (CIE) lab parameters for PI film (a thickness of 20 μm). D65 standard illuminant and a field view of 10° were used [18]. Chroma (C*) was calculated by the values of a* and b* through the following equation:

$$C^{*}=\sqrt{{a^{*}}^{2}+{b^{*}}^{2}}$$

where a* and b* are coordinates denoting the color components of green, red, blue, and yellow. The tested variable L* denotes the color lightness. 

The Gaussian 16 package was used for the theoretical calculation of molecular energy levels and geometries. Using 6-311G(d,p) on all atoms, the ground-state geometries of the DPPTENFPDA and DPPTENFPPI units were thoroughly optimized at the DFT level by the three-parameter Becke functional and the correlation functional of B3LYP. To guarantee that the optimized molecular geometries had the lowest total energy, the vibration frequencies were computed analytically. By using B3LYP/6-311++G(d,p), the transition energy and oscillator strength for the lowest 25 transitions of the DPPTENFPDA and DPPTENFPPI units were determined via TDDFT on the optimized ground-state geometries. The polarizable continuum model was used for establishing solvation influence.

### 2.3. Preparation of the Monomer

Synthesis of 2,5-bis(2-ethylhexyl)-3,6-di(thiophen-2-yl)-2,5-dihydropyrrolo

[3,4-c]pyrrole-1,4-dione (DPPTEN)

TDPP (6.000 g, 20 mmol), 2-ethylhexyl bromide (19.312 g, 100 mmol), potassium carbonate (11.0568 g, 80 mmol), and DMF (100 mL) were added to a flask and agitated for 24 h at 110 °C. Then, the solution was filtered with a Buchner funnel to eliminate the salt byproduct. The product was concentrated and purified through silica-gel column chromatography with n-hexane/dichloromethane (*v*/*v* = 1/2). Finally, a dark red product was obtained. ^1^H NMR (400 MHz, Chloroform-*d*, *δ*, ppm) 8.82 (dd, *J* = 3.9, 1.2, 2H), 7.55 (dd, *J* = 5.0, 1.2, 2H), 7.19 (d, *J* = 9.0, 2H), 4.02–3.88 (m, 4H), 1.78 (h, *J* = 7.0, 6.5, 2H), 1.22 (dddd, *J* = 36.3, 15.3, 8.4, 4.3, 16H), 0.79 (dt, *J* = 9.0, 7.1, 12H).^13^C NMR (100 MHz, Chloroform-*d*, *δ*, ppm) 161.75, 140.42, 135.29, 130.53, 129.84, 128.42, 107.92, 45.85, 39.08, 30.22, 30.20, 28.36, 23.54, 23.06, 14.02, 10.49. MS (EI, m/z): 524.48 ([M]^+^, calcd for C_30_H_40_N_2_O_2_S_2_, 524.25). Anal. Calcd for C_30_H_40_N_2_O_2_S_2_: C, 68.66; H, 7.68; N, 5.34; S, 12.22; Found: C, 68.58; H, 7.71; N, 5.33; S, 12.26.

Synthesis of 3,6-bis(5-bromothiophen-2-yl)-2,5-bis(2-ethylhexyl)-2,5-

dihydropyrrolo[3,4-c]pyrrole-1,4-dione (DPPTENBr)

DPPTEN (0.525 g, 1 mmol) and chloroform (50 mL) were added to a flask and agitated at 0 °C for 30 min, avoiding light. *N*-bromosuccinimide (0.373 g, 2.1 mmol) was added with agitation for 30 min. The solution was then filtered. The organic phase was cleaned and dried by water and MgSO_4_, respectively. Then, the solvent was extracted at low pressure. Silica-gel column chromatography was used to purify the product with n-hexane/dichloromethane (*v*/*v* = 1/1). Finally, a purple-black product was obtained. ^1^H NMR (400 MHz, Chloroform-*d*, *δ*, ppm) 8.61 (d, *J* = 4.2, 2H), 7.18 (d, *J* = 4.2, 2H), 3.95–3.83 (m, 4H), 1.79 (h, *J* = 6.8, 6.3, 2H), 1.35–1.19 (m, 16H), 0.85 (q, *J* = 7.1, 12H).^13^C NMR (100 MHz, Chloroform-*d*, *δ*, ppm) 161.32, 139.35, 135.41, 131.44, 131.16, 119.05, 107.93, 45.98, 39.08, 30.16, 28.32, 28.30, 23.55, 23.05, 14.04, 10.46. MS (EI, m/z): 682.22 ([M]^+^, calcd for C_30_H_38_Br_2_N_2_O_2_S_2_, 680.07). Anal. Calcd for C_30_H_38_Br_2_N_2_O_2_S_2_: C, 52.79; H, 5.61; N, 4.10; S, 9.39; Found: C, 52.81; H, 5.58; N, 4.13; S, 9.35.

Synthesis of 3,6-bis[5-(4-amino-3-fluorophenyl)thiophen-2-yl]-2,5-bis

(2-ethylhexyl)pyrrolo[4,3-c]pyrrole-1,4-dione (DPPTENFPDA)

DPPTENBr (1.024 g, 1.5 mmol) and 4-amino-3-fluorophenylboronic acid pinacol ester (0.711 g, 3 mmol) were dissolved in 150 mL of THF. Six drops of Aliquat 336 and 4.5 mL of 2M aqueous K_2_CO_3_ solution were added. After that, the solution was kept at 75 °C with stirring under argon. Subsequently, the catalyst Pd[P(C_6_H_5_)_3_]_4_ was added and agitated for 36 h. The diamine was purified by flash chromatography (2.5% ethyl acetate in CH_2_Cl_2_) after it had cooled. Finally, a black product was obtained. ^1^H NMR (400 MHz, Chloroform-*d*, *δ*, ppm) 8.32 (d, *J* = 3.8, 2H), 7.25 (t, *J* = 10.5, 4H), 6.77–6.61 (m, 4H), 4.14–3.80 (m, 8H), 1.83 (q, *J* = 6.8, 2H), 1.23 (ddd, *J* = 42.5, 12.5, 6.5, 16H), 0.82–0.60 (m, 12H). ^13^C NMR (100 MHz, Chloroform-*d*, *δ*, ppm) 161.76, 152.64, 149.26, 139.64, 136.84, 135.46, 135.33, 127.68, 124.03, 123.22, 122.63, 122.60, 116.93, 116.89, 113.18, 112.98, 107.97, 45.98, 39.24, 30.39, 28.62, 23.73, 23.13, 14.08, 10.61. MS (EI, m/z): 743.3240 ([M]^+^, calcd for C_42_H_48_F_2_N_4_O_2_S_2_, 743.3260). Elem. Anal.: Calcd for C_42_H_48_F_2_N_4_O_2_S_2_: C, 71.35; H, 7.13; N, 7.92; S, 9.07; Found: C, 71.66; H, 7.08; N, 7.86; S, 9.06. 

### 2.4. Preparation of Polyimides

TDPP-containing polyimide (DPPTENFPPI) was obtained via the polycondensation reaction between DPPTENFPDA and PMDA, using the thermal imidization method. The process was as follows: DPPTENFPDA (0.8414 g, 1.13 mmol) was added to NMP (6 mL). Subsequently, PMDA (0.2465 g, 1.13 mmol) was placed into the solution. After stirring with an ice bath for 8 h under a nitrogen atmosphere, a viscous PAA solution was formed, which was applied in an even coating on a glass pane. The PAA film was thermally imidized by vacuum heating (1 h at 100 °C, 1 h at 200 °C, 1 h at 300 °C, and 0.5 h at 380 °C) to yield DPPTENFPPI. IR (*v*, cm^−1^): 1734 and 1778 cm^−1^ (stretching of C=O), 1373 (stretching of C-N). Furthermore, a Kapton film was also prepared. Using a similar process to that described above, PMDA was reacted with ODA to produce PAA. After coating a glass plate with the PAA, it was thermally imidized to produce a Kapton film by vacuum heating at 100 °C (1 h)/100~200 °C (1 h)/200~300 °C (1 h)/300~370 °C (1 h).

### 2.5. Preparation of Two-Layer FCCL (2L-FCCL)

The PAA solution was doctor-bladed on a copper foil with a thickness of 18 μm. Then, it was thermally imidized by vacuum heating at 100 °C (1 h)/200 °C (1 h)/300 °C (1 h)/380 °C (0.5 h) to yield the corresponding DPPTENFPPI-based 2L-FCCL.

## 3. Results and Discussion

### 3.1. Preparation and Characterization of Monomers

**Figure 1:**
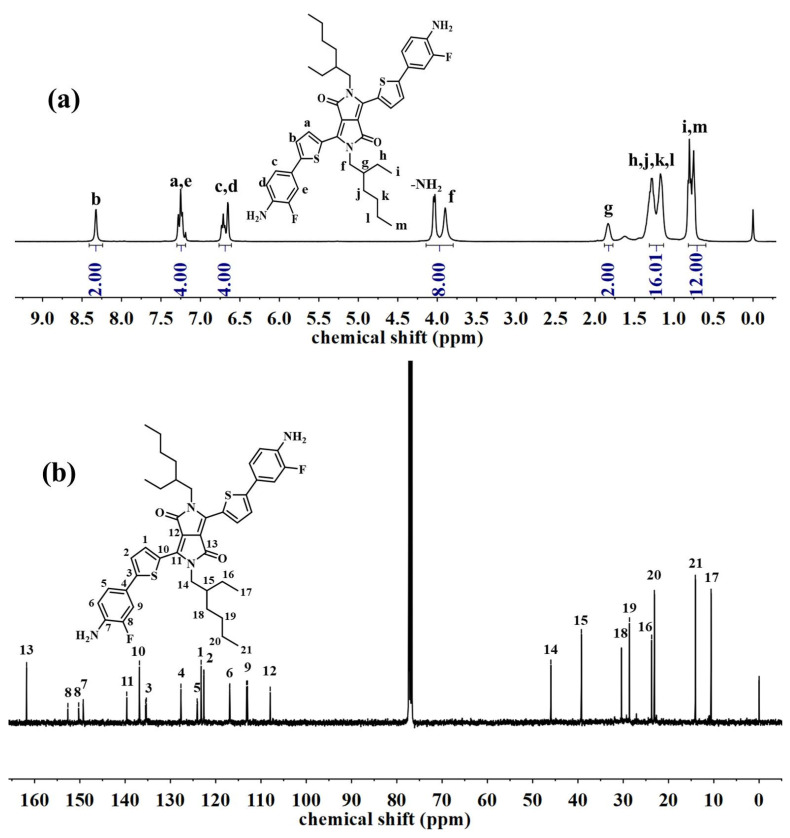
^1^H NMR (**a**) and ^13^C NMR (**b**) spectra of DPPTENFPDA in CDCl_3_.

As outlined in Scheme 1, the synthesis of DPPTENFPDA is accomplished by a three-stage procedure. The structures of DPPTENFPDA and the corresponding intermediate monomers were verified via NMR (Figure 1, Figure S2 and Figure S3), MS analysis (Figures S4–S6), FTIR (Figure S7), and elemental analyses. The analytical findings validate the successful synthesis of DPPTENFPDA, DPPTENBr, and DPPTEN.

### 3.2. Synthesis and Characterizations of DPPTENFPPI

Working according to Scheme S1, DPPTENFPPI was synthesized through a classic two-step procedure. The DPPTENFPDA reacted with PMDA to generate poly(amic acid) (DPPTENFPPAA). Then, it was thermally imidized to form DPPTENFPPI. According to the GPC results, DPPTENFPPAA has a high weight average molecular weight (*M_w_*) of 1.15 × 10^5^ g/mol, and the polydispersity index is 1.96. The chemical structure of DPPTENFPPI was verified by an FTIR spectrogram (Figure S7). In the spectrum of DPPTENFPPI, the peaks at 1734 cm^−1^ and 1778 cm^−1^ are related to the carbonyl (C=O) bond stretching, while the peak at 1373 cm^−1^ is indicative of the C-N bond stretching [19,20,21]. Meanwhile, the absorption bands of amine groups (1619 cm^−1^ and 3358 cm^−1^) in DPPTENFPDA have vanished in the spectrogram of DPPTENFPPI. These analyses support the successful preparation of DPPTENFPPI.

### 3.3. Optical Properties and Theoretical Calculation

**Figure 2:**
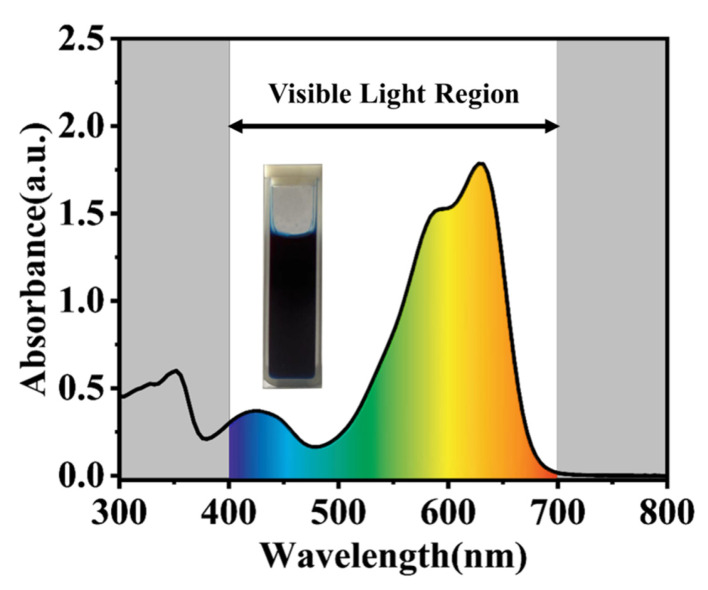
UV-Vis absorption spectrums of DPPTENFPDA in a DMF solution (the colored area indicates visible-light absorption. Inset: Photograph of a 5 μM DPPTENFPDA solution in DMF).

Figure 2 displays the UV/vis absorption profile of the DPPTENFPDA solution. It exhibits intense and broad absorption across the entire visible range. Within this range, two distinct bands are observed: a prominent band concentrated at 628 nm, characterized by a full width at half-maximum (FWHM) of around 102 nm, and another band at 424 nm. An additional peak was noted at 352 nm. The DPPTENFPDA molecule bears a chromophore system composed of a TDPP core connected to two flanking fluorinated benzene rings. The structural planarity and high conjugation of the TDPP-based chromophore broaden and red-shift the visible absorption profile of DPPTENFPDA, thereby producing hyperchromic effects. As illustrated in the inset of Figure 2, a dilute DPPTENFPDA solution appears deep blue-black, which is primarily attributable to its structural attributes, broad FWHM in the visible range, and low absorption in the blue region. The detailed assignments of electronic transitions corresponding to the observed absorption of DPPTENFPDA will be further explored via theoretical analysis in the subsequent section. 

**Figure 3:**
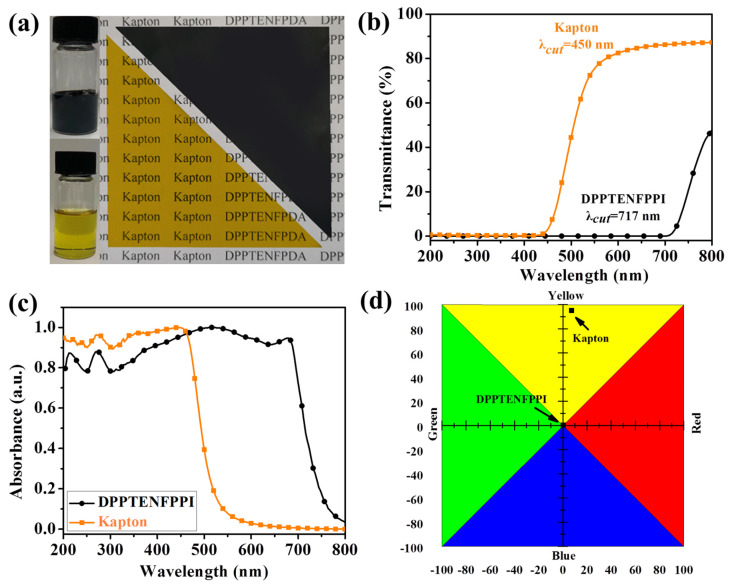
(**a**) The photo images of PAA solutions and PI films with a thickness of 20 μm (DPPTENFPPI (black); Kapton (yellow)); (**b**) transmission and (**c**) absorption UV−vis spectra for DPPTENFPPI and Kapton films; (**d**) CIE lab color parameters.

The optical performance of the DPPTENFPPI film was also examined. Figure 3a shows the images of the DPPTENFPPI film and the associated PAA solution. For comparative purposes, the Kapton film is also shown. Notably, the Kapton film exhibits high transparency and yellow coloration. In contrast, with the incorporation of a chromophore based on the TDPP moiety, the DPPTENFPPI film displays a deep black color with high opacity, indicating its exceptional masking properties. The UV/visible transmission and absorption spectrograms of the two PIs are displayed in Figure 3b,c. Table 1 presents the relevant data. The Kapton film demonstrates a *λ_cut_* of 450 nm, while DPPTENFPPI achieves a high *λ_cut_* of 717 nm, presenting a redshift of 267 nm compared with the Kapton film. Furthermore, the *λ_cut_* of DPPTENFPPI displays a redshift of 162 nm in relation to the BPI published by Liu (NDA-PMDA, *λ_cut_* = 555 nm) [15] and a redshift of 33 nm in relation to the BPI in our previous research (TPCPFPPI, *λ_cut_* = 684 nm) [14]. The onset absorption wavelength (*λ_onset_^abs^*) of Kapton films is 521 nm, whereas that of DPPTENFPPI is higher, at 780 nm. Evidently, DPPTENFPPI possesses a red-shifted and wide absorption profile over the full visible-spectrum region. The light transmittance at 700 nm (T_700_) of Kapton films is 86%, while that of DPPTENFPPI drops to 0%, demonstrating the highly opaque nature of DPPTENFPPI film. 

The CIE-Lab system is commonly utilized for color characterization [14]. L* denotes the color lightness, which changes from 0 to 100. The color components of red, green, yellow, and blue are represented by the coordinates of a* and b*. Chroma (C*) is derived from coordinate a* and coordinate b*. Lower C* and L* levels indicate an intensified black hue [22]. Figure 3d and Table 1 present the CIE results. The Kapton film has L* and C* values of 71.63 and 95.58, but those of the DPPTENFPPI film significantly fall to 0.86 and 0.39, approaching pure black (C* = L* = 0). Moreover, the DPPTENFPPI film shows a more profound black coloration in relation to the other published BPIs, encompassing commercially available carbon black-doped PIs (BY122 and BY112) and other inherently black PIs (Table 1). These results confirm the effectiveness of a chromophore based on the TDPP moiety in achieving an inherent black color for PIs. With the incorporation of TDPP connected with fluorine-substituted benzene rings, the DPPTENFPPI film demonstrates much wider absorption and reduced optical transmittance, thereby showing a black appearance and heightened opaqueness.

**Figure 4:**
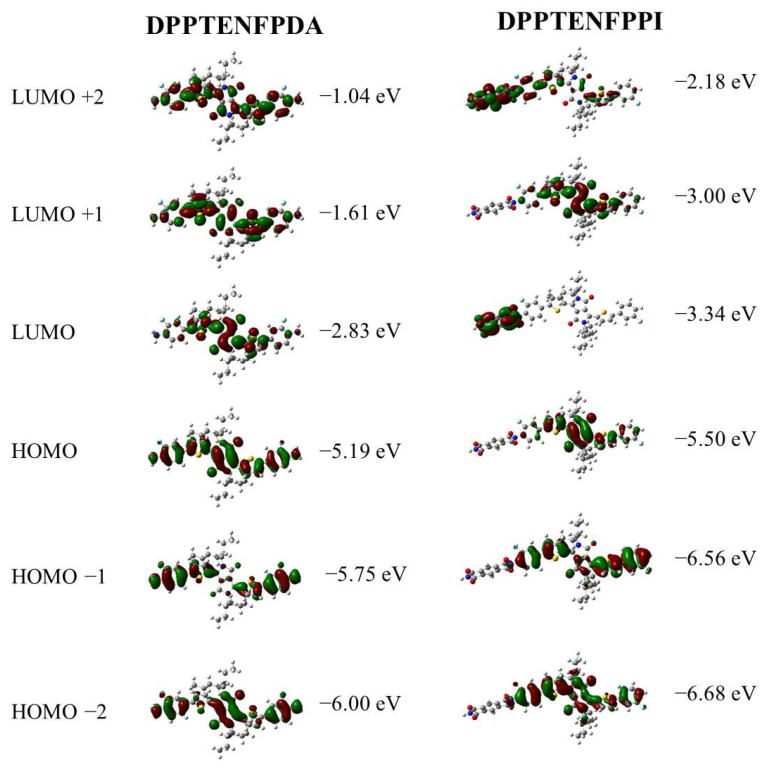
The frontier molecular orbitals of the DPPTENFPDA and DPPTENFPPI units.

The frontier molecular orbitals (FMOs) of both the DPPTENFPDA and DPPTENFPPI units were analyzed with the Gaussian 09 (Revision D.01) software [14,23]. Visual representations of the FMO distribution are presented in Figure 4. In the case of DPPTENFPDA, the FMOs are extensively dispersed across the entire molecular framework. Conversely, within the DPPTENFPPI unit, the HOMOs and LUMO + 1 orbitals are predominantly assigned to the diamines, while the LUMO and LUMO + 2 orbitals are prominently distributed within the dianhydrides. The FMOs of the DPPTENFPPI demonstrate a reduced energy level compared to those of the DPPTENFPDA, which can be explained by the electron-accepting action of imide [24].

**Figure 5:**
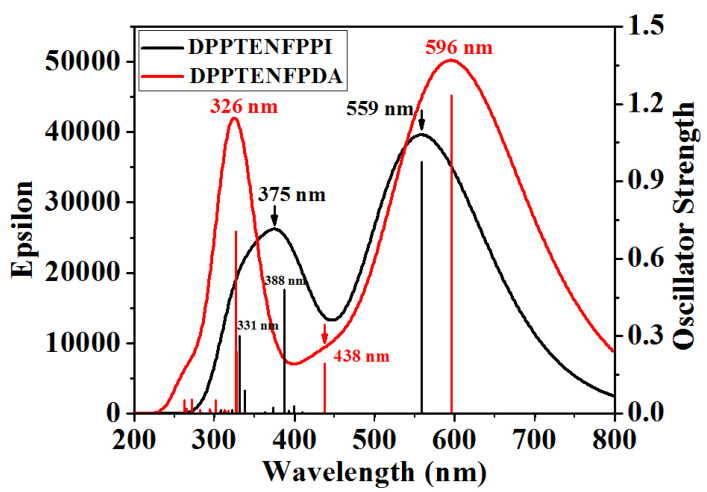
UV-Vis absorption spectra of the DPPTENFPDA and DPPTENFPPI units as predicted by the TD-DFT method, with oscillator strengths marked by vertical lines.

The study of the light-absorption properties of DPPTENFPDA and DPPTENFPPI was conducted by utilizing TD-DFT with the B3LYP/6-311++G(d,p) basis set [25,26,27]. A polarizable continuum model was used for establishing solvation influence. The absorption spectra simulated by TD-DFT are depicted in Figure 5, with the detailed transition data listed in Table 2. In the calculated absorption profile of DPPTENFPDA, a wide and significant peak can be observed at 596 nm, accompanied by a weak peak at 438 nm within the visible range (as seen in Figure 5). An additional peak is visible at 326 nm. The overall light absorption features of DPPTENFPDA are in accordance with the experimental spectrum presented in Figure 2. Hence, the observed peak at 628 nm in DPPTENFPDA can be attributed to the S1 state formation associated with the HOMO to LUMO electronic transition (99.8%). The experimental peak at 424 nm in DPPTENFPDA results from the electronic transition of S0 → S3, which is associated with the HOMO-2 to LUMO electronic transition (96.4%). The peak at 352 nm is due to these higher-energy transitions, with the transition of S0 → S9 displaying the most significant oscillator strength (f = 0.7075). In the calculated absorption profile of DPPTENFPPI, the wide visible peak at 559 nm is correlated with S2 state generation, primarily involving the HOMO→LUMO + 1 (99.7%) transition. The wide band centered at 375 nm mainly consists of two absorption bands. One band is at 388 nm, involving the S0 → S8 transition. The other is at 331 nm, involving the S0 → S18 transition. Compared to DPPTENFPDA, DPPTENFPPI exhibits a blue-shifted spectrum, which is primarily ascribable to the electron-accepting characteristics of the imide [28,29].

**Figure 6:**
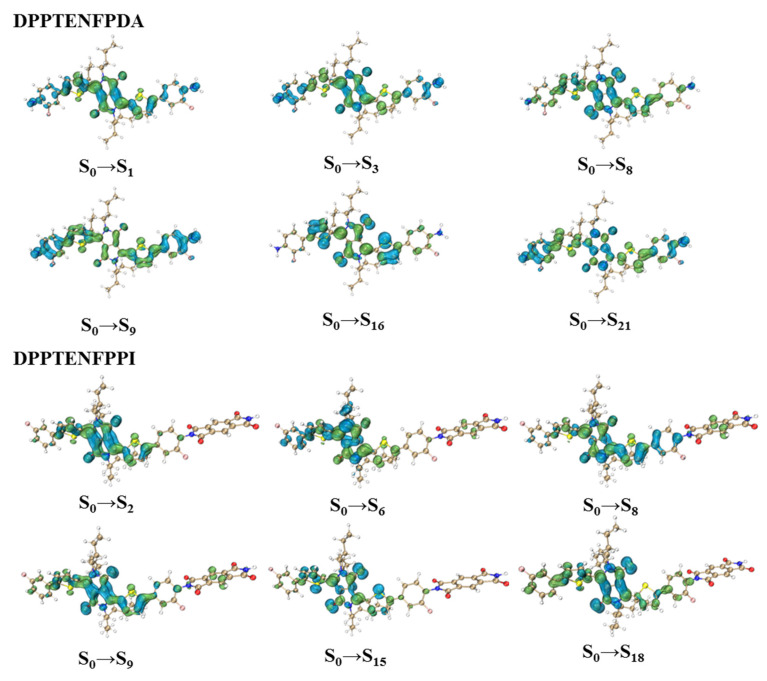
The electronic nature of S_n_ excitation in the DPPTENFPDA and DPPTENFPPI units based on hole and electron distribution, with the charge transfer from blue to green.

To better understand the characteristics of electron transition, the hole–electron distribution was studied using the Multiwfn (3.8 dev bin Win64) software [30]. Figure 6 illustrates the hole and electron distributions for the S0 → Si transitions in DPPTENFPDA and DPPTENFPPI. The green and blue isosurfaces stand for the distributions of electrons and holes, respectively. The high blackness of DPPTENFPPI is primarily due to its strong visible absorption, which is predominantly attributable to the transition of S0 to S2. In this S0 → S2 transition for DPPTENFPPI, the electrons and holes are distributed across the chromophore, with a particular concentration on the TDPP segment, as depicted in Figure 6. Similarly, the absorption in the visible spectrum of DPPTENFPDA is mainly derived from the transition of S0 to S1, which exhibits charge migration features akin to those in the S0 → S2 transition of DPPTENFPPI. Both transitions, the S0 to S1 in DPPTENFPDA and S0 to S2 in DPPTENFPPI, are related to the π−π* transitions, mainly owing to localized excitation [31,32]. To sum up, the theoretical analysis indicates that the profound darkness of DPPTENFPPI is largely owing to the HOMO → LUMO + 1 transition within the chromophore moiety.

### 3.4. Electrical Performances

The electrical performance of the DPPTENFPPI and Kapton films are assessed and presented in Table 3. DPPTENFPPI demonstrates a dielectric strength of 275 kV/mm, along with a volume resistivity of 7.86 × 1014 Ω·m and a surface resistivity of 8.32 × 1014 Ω. The electrical characteristics of DPPTENFPPI are found to be comparable with those of Kapton film. The application of filler-doped BPI composites is significantly constrained due to reduced electrical performance [7]. However, DPPTENFPPI has superior electrical performance compared to the filler-doped BPI composites [33,34,35], as presented in Table 3. The high electrical performance of DPPTENFPPI is predominantly attributed to its lack of black filler [36,37,38], which ensures its potential utilization in microelectronics, such as in the flexible printed circuit board (FPCB) industry [3,7].

### 3.5. Thermal Performance

**Figure 7:**
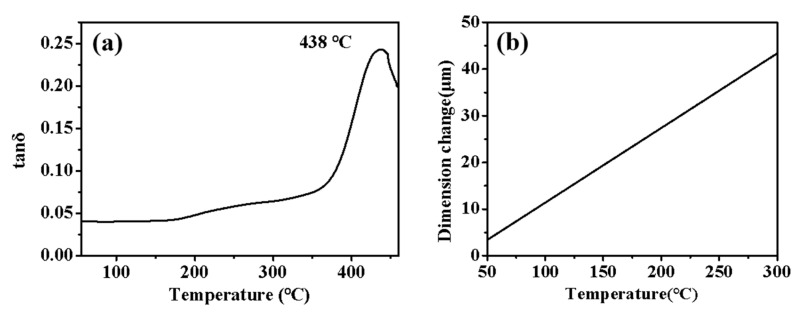
DMA (**a**) and TMA (**b**) curves of the DPPTENFPPI film.

The thermal characteristics of DPPTENFPPI were assessed through DMA and TMA, as shown in Figure 7. DPPTENFPPI exhibits a glass transition temperature (*T_g_*) of 438 °C (Figure 7a). Furthermore, DPPTENFPPI demonstrates excellent dimensional stability, as indicated by its notably low CTE of 16 ppm/K between 50 °C and 300 °C (Figure 7b). The CTE of DPPTENFPPI is significantly lower than the commercially available carbon black-enriched BPI, which has a CTE of 60 ppm/K [7], and is also lower than other reported graphite-enriched BPIs with a CTE of 40 ppm/K [41]. Obviously, DPPTENFPPI exhibits a CTE that closely matches that of the copper foil layer (CTE = 18 ppm/K). Such high matching of CTE is advantageous for enhancing the dependability of the final FCCLs. The reduced CTE observed in DPPTENFPPI mainly stems from its inherent structural features. DPPTENFPDA and PMDA possess planar rigid structures, which result in a comparatively linear and flat backbone for DPPTENFPPI. Additionally, the lactam units within DPPTENFPPI can induce dipole–dipole interactions, thereby enhancing the intermolecular forces [42]. These factors promote dense chain packing, consequently leading to the observed low CTE [43,44]. The film surface and section morphologies of DPPTENFPPI were examined by SEM. The results are shown in Figure S8. It can be seen that the film section is compact and the film surface is flat and smooth, demonstrating the high quality of the PI film.

### 3.6. Performance of DPPTENFPPI-Based 2L-FCCL

To manufacture 2L-FCCL, the DPPTENFPPAA was applied to copper foil layers without a resin adhesive layer being used. Subsequently, the DPPTENFPPAA was thermally imidized to form a DPPTENFPPI/Cu laminate. The solder heat resistance and peeling strength of the DPPTENFPPI/Cu laminate were examined and are shown in Table 4. The DPPTENFPPI/Cu laminate presents a high peeling strength of 0.81 N/mm, which is mostly explained by the existence of lactam groups [45,46]. Additionally, the DPPTENFPPI/Cu laminate exhibits remarkable resistance to solder heat as a result of the exceptional thermal stability of DPPTENFPPI. Even in a solder bath of 350 °C, the DPPTENFPPI/Cu laminate does not show delamination and blistering.

## 4. Conclusions

In summary, a diamine (DPPTENFPDA) derived from TDPP-based chromophores was synthesized. The polymerization of DPPTENFPDA with PMDA yielded a high-performance black polyimide (DPPTENFPPI). The optical performance results demonstrated that DPPTENFPPI achieved whole visible absorption with a large *λ_cut_* of 717 nm and a small L* of 0.86. Computational studies indicated that the profound blackness of DPPTENFPPI chiefly arose from the transition of HOMO→LUMO + 1 within the chromophore system. Furthermore, DPPTENFPPI exhibited outstanding electrical and thermal performance while maintaining a low CTE. The DPPTENFPPI-based 2L-FCCL presents high solder heat resistance and peeling strength. This work offers vital insights for the advancement of BPIs with excellent overall properties for microelectronics applications.

## Figures and Tables

**Table 1:** Comparison of the optical properties of the DPPTENFPPI and Kapton films with other BPI films reported in the literature.

PIs	*λ_cut_* ^a^ (nm)	T_700_ ^b^ (%)	$\lambda_{onset}^{abs}$ (nm)	L*	a*	b*	C*	Reference
DPPTENFPPI	717	0	780	0.86	0.34	0.20	0.39	this work
Kapton	450	86	521	71.63	7.2	95.31	95.58	this work
TPCPFPPI	684	>15	747	1.33	0.39	−0.18	0.43	[14]
BY112	-	-	-	22.7	0.3	−2.7	2.72	[7]
BY122	-	-	-	21.9	0.3	−2.6	2.62	[7]
PI-2	-	>40	-	28.4	−3.40	5.40	6.38	[7]
PI-4	-	>12	-	23.1	0.3	−2.1	2.12	[7]
PI-6	-	>8		21.5	0.5	−2.2	2.26	[7]
PI-5	555	>55	-	23.36	46.43	40.26	61.45	[15]
PI-a	592	>15	-	2.2	11.99	3.33	12.36	[3]
PI-b	578	>21	-	4.23	23.67	7.26	24.76	[3]

^a^ Cutoff wavelength of the films; ^b^ transmittance at a wavelength of 700 nm.

**Table 2:** The transition energy (E), transition wavelength (λ), oscillator strength (f), and electronic configuration involved in the transitions for DPPTENFPDA and DPPTENFPPI units.

Excited State	E (eV)	λ (nm)	f ^a^	Electronic Configuration ^b^
DPPTENFPDA				
1	2.08	596	1.2332	H → L (99.8%)
3	2.83	438	0.1935	H-2 → L (96.4%)
8	3.79	327	0.2407	H → L + 2 (57.1%)
9	3.80	326	0.7075	H-1 → L + 1 (67.3%), H → L + 2 (12.1%)
16	4.11	302	0.0529	H-11 → L (63.6%), H-9 → L (12.1%)
21	4.56	272	0.055	H-2 → L + 2 (82.5%), H-3 → L + 1 (11%)
DPPTENFPPI				
2	2.22	559	0.9761	H → L + 1 (99.7%)
6	3.11	399	0.0288	H-1 → L + 1 (46.7%), H-3 → L + 1 (41.5%)
8	3.20	388	0.4815	H-2 → L + 1 (79.2%)
9	3.32	374	0.0243	H → L + 3 (53.9%), H-1 → L + 1 (18.9%)
15	3.66	338	0.0892	H-6 → L + 1 (42.5%), H → L + 5 (29.6%)
18	3.75	331	0.3008	H → L + 5 (61.4%), H-6 → L + 1 (16.5%)

^a^ Transitions where f ˃ 0.02 are listed; ^b^ transitions with a contribution of ≥ 10% are listed.

**Table 3:** Comparison of the electrical properties of the DPPTENFPPI and Kapton films with other BPI composite films reported in the literature.

Polyimide	Surface Resistivity (Ω)	Volume Resistivity (Ω·m)	Dielectric Strength (kV/mm)	Reference
DPPTENFPPI	8.32 × 10^14^	7.86 × 10^14^	275	In this work
Kapton	2.85 × 10^14^	3.66 × 10^14^	286	In this work
SF315C (composite)	4.48 × 10^11^	5.89 × 10^13^	93.3	[39]
PI-0 (composite)	-	-	89	[40]
PI-A 6%CB (composite)	8.16 × 10^8^	-	-	[33]
PI-B 6%CB (composite)	1.03 × 10^9^	-	-	[33]
PI/0.5%SRGO (composite)	-	1.1 × 10^6^	-	[34]

**Table 4:** The properties of the 2L-FCCL.

FCCL	Peeling Strength (N/mm)	Solder Heat Resistance (350 °C)
DPPTENFPPI/Cu	0.81	Good ^a^

^a^ No blistering and delamination under a solder bath at 350 °C.

## Data Availability

The original contributions presented in the study are included in the article/Supplementary Materials.

## Supplementary Materials

The following are available online at https://www.mdpi.com/article/10.3390/polym16233365/s1: Figure S1. Applications of Kapton and BPI films in flexible copper clad laminates. Figure S2. The 1H NMR (a) and 13C NMR (b) spectra of DPPTEN. Figure S3. The 1H NMR (a) and 13C NMR (b) spectra of DPPTENBr. Figure S4. The MS spectrum of DPPTEN. Figure S5. The MS spectrum of DPPTENBr. Figure S6. The MS spectrum of DPPTENFPDA. Scheme S1. Synthesis route of DPPTENFPPI. Figure S7. FT-IR spectra of intermediates, diamine and DPPTsENFPPI. Figure S8. SEM images of film surface morphology (a) and film section morphology (b) for DPPTENFPPI.

## Abbreviations

BPI	black polyimide
FCCLs	flexible copper-clad laminates
TDPP	3,6-bis(thiophen-2-yl)diketopyrrolopyrroles
PMDA	pyromellitic dianhydride
λ_cut_	cut-off wavelength
CTE	coefficient of thermal expansion
HOMO	highest occupied molecular orbital
LUMO	lowest unoccupied molecular orbital
PIs	polyimides
CTCs	charge transfer complexes
TPCP	tetraphenylcyclopentadienone
ODA	4,4′-oxydianiline
THF	Tetrahydrofuran
DMF	N, N-dimethylformamide
NMP	1-methyl-2-pyrrolidinone
NMR	nuclear magnetic resonance
CDCl_3_	deuterated chloroform
TMS	tetramethylsilane
MS	mass spectrometry
FTIR	Fourier-transform infrared spectroscopy
DMA	dynamic mechanical analysis
TMA	thermomechanical analysis
CIE	Commission International Eclairage
DPPTEN	2,5-bis(2-ethylhexyl)-3,6-di(thiophen-2-yl)-2,5-dihydropyrrolo[3,4-c]pyrrole-1,4-dione
DPPTENBr	3,6-bis(5-bromothiophen-2-yl)-2,5-bis(2-ethylhexyl)-2,5-dihydropyrrolo[3,4-c]pyrrole-1,4-dione
DPPTENFPDA	3,6-bis [5-(4-amino-3-fluorophenyl)thiophen-2-yl]-2,5-bis(2-ethylhexyl)pyrrolo[4,3-c]pyrrole-1,4-dione
PAA	poly (amic acid)
FWHM	full width at half-maximum
λ_onset_^abs^	onset absorption wavelength
T_700_	light transmittance at 700 nm
FMOs	frontier molecular orbitals
FPCB	flexible printed circuit board
