# Supplementary material for: Synthesis and Characterization of Polyimide with High Blackness and Low Thermal Expansion by Introducing 3,6-bis(thiophen-2-yl)diketopyrrolopyrrole-Based Chromophores
Source: Polymers (Basel). 2024 Nov 29;16(23):3365. doi: 10.3390/polym16233365 (PMC11644352; doi:10.3390/polym16233365)
Supplement: Supplementary file 1 [file polymers-16-03365-s001.zip › polymers-3282095-supplementary.pdf]

Supporting Information

Synthesis and Characterization of Polyimide with High Blackness and Low Thermal Expansion by Introducing 3,6-bis(thiophen-2-yl) diketopyrrolopyrrole-Based Chromophores

Yiwu Liu, Xueyuan Liu, Jinghua Tan *, Jie Huang *, Jiazhen Yuan, Huipeng Li, Jieping Guo, Penghao Yu and Yue Chen

National and Local Joint Engineering Research Center for Advanced Packaging Materials and Technology, Key Laboratory of Advanced Packaging Materials and Technology of Hunan Province, School of Packaging and Materials Engineering, Hunan University of Technology, Zhuzhou 412007, China

* Correspondence: tjh@hut.edu.cn (J.T.); huangjie3@alumni.sjtu.edu.cn (J.H.)

Table of Contents

Figure S1 3

Figure S2 3

Figure S3 4

Figure S4 4

Figure S5 5

Figure S6 5

Scheme S1 6

Figure S7 7

Figure S8 7

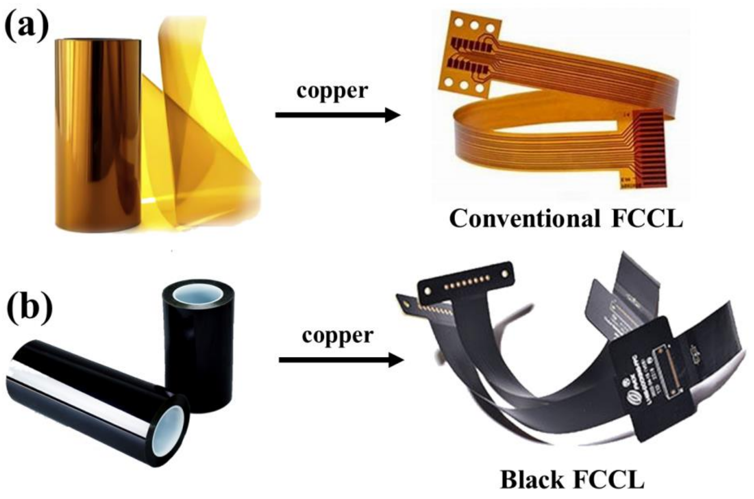

Figure S1. Applications of Kapton and BPI films in flexible copper clad laminates.

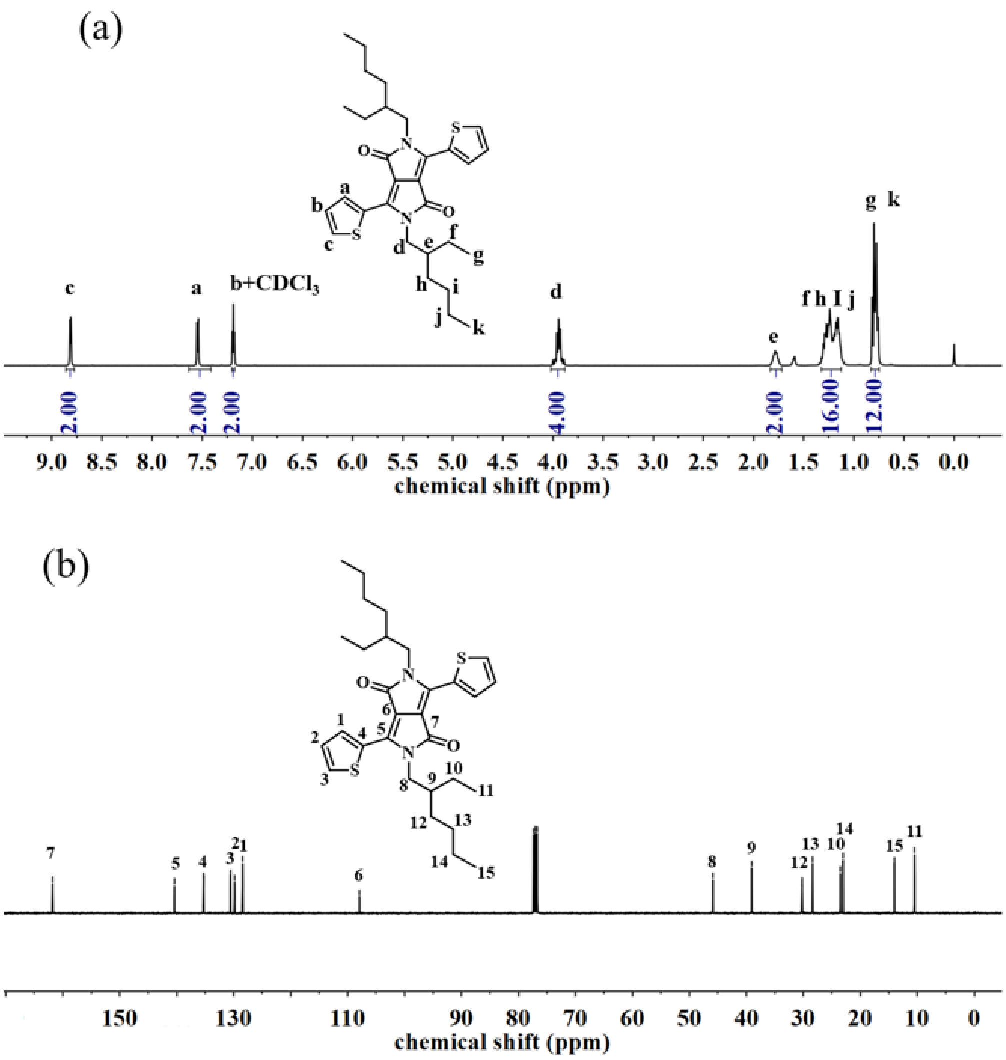

Figure S2. The ^{1}H NMR (a) and ^{13}C NMR (b) spectra of DPPTEN.

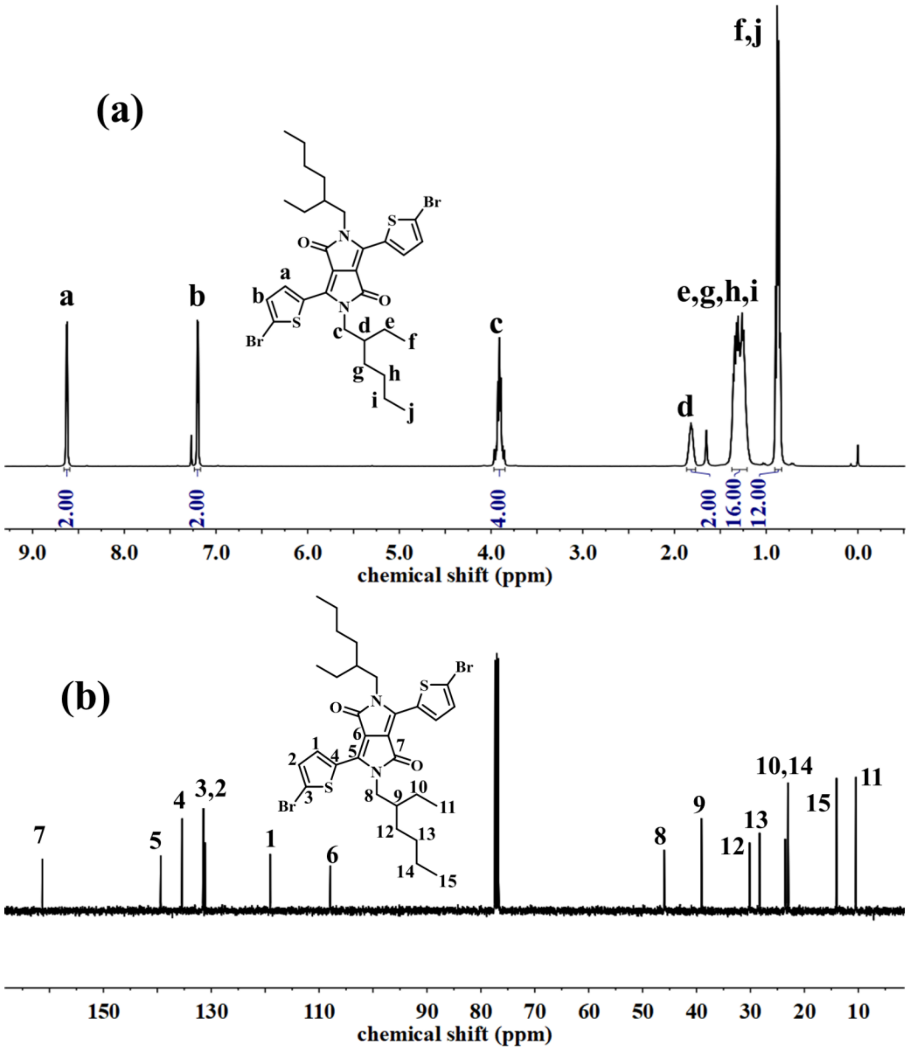

Figure S3. The ^{1}H NMR (a) and ^{13}C NMR (b) spectra of DPPTENBr.

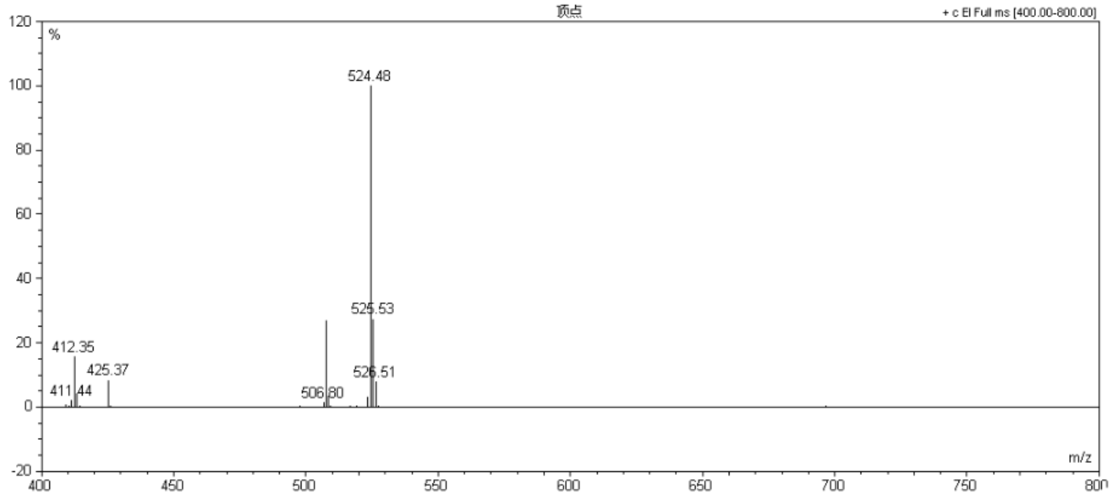

Figure S4. The MS spectrum of DPPTEN.

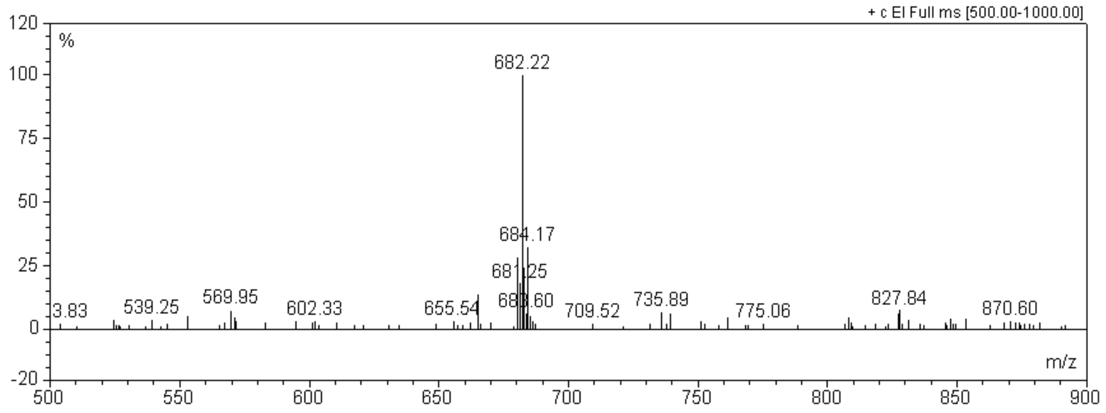

Figure S5. The MS spectrum of DPPTENBr.

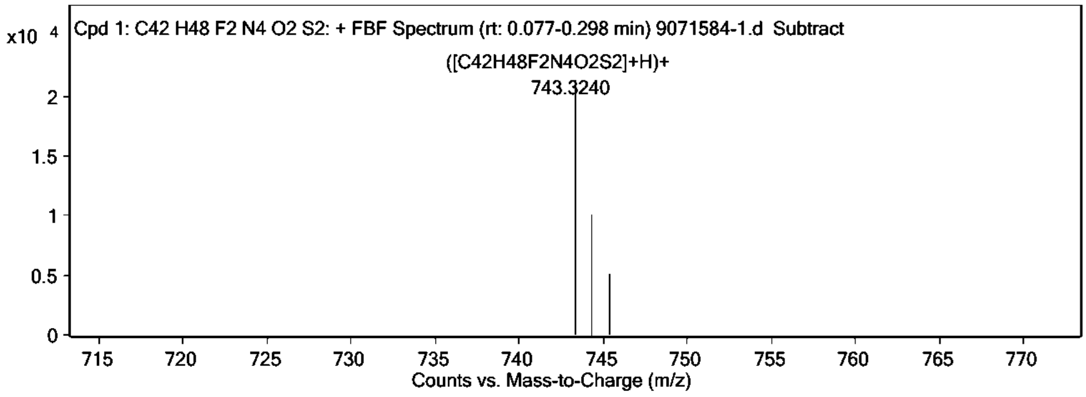

Figure S6. The MS spectrum of DPPTENFPDA.

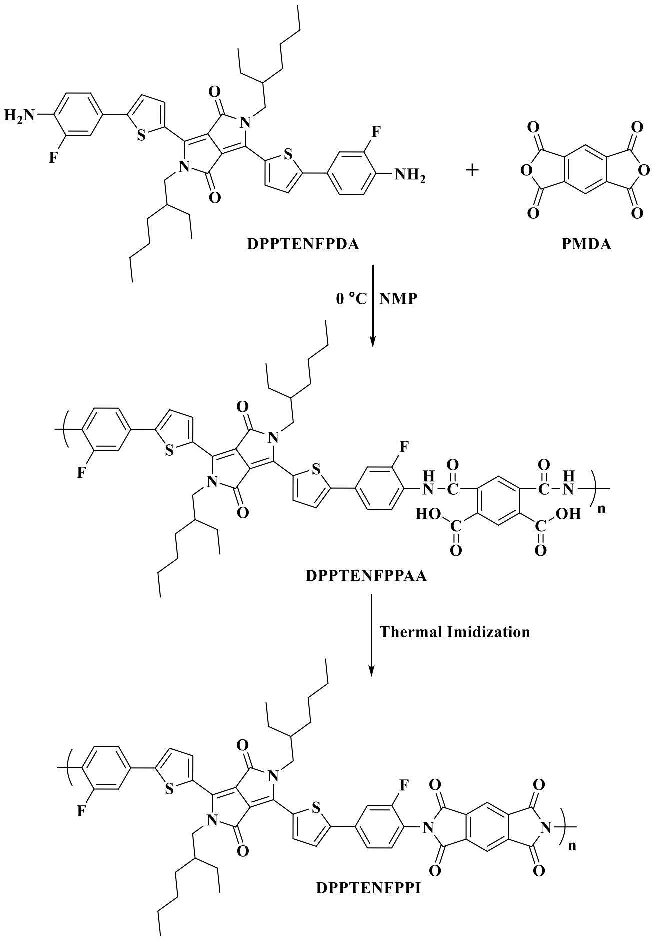

Scheme S1. Synthesis route of DPPTENFPPI.

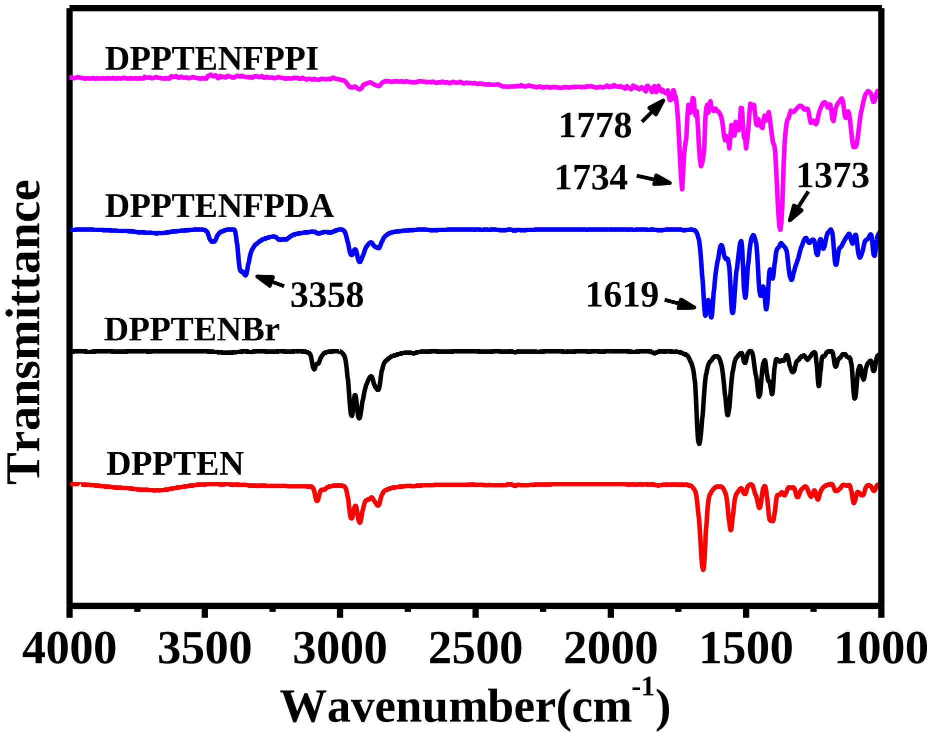

Figure S7. FT-IR spectra of intermediates, diamine and DPPTENFPPI.

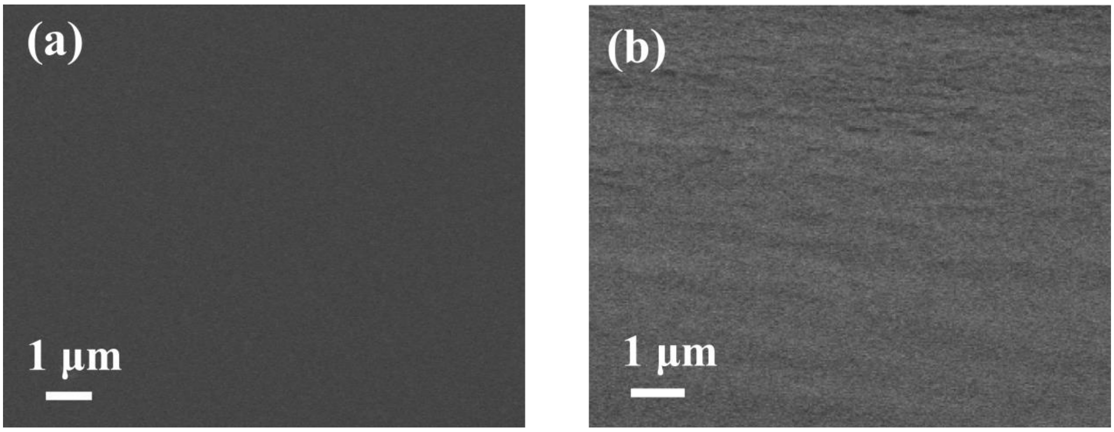

Figure S8. SEM images of film surface morphology (a) and film section morphology (b) for DPPTENFPPI.
